# Healthcare managers in negative media focus: a qualitative study of personification processes and their personal consequences

**DOI:** 10.1186/1472-6963-14-8

**Published:** 2014-01-07

**Authors:** Maria Wramsten Wilmar, Gunnar Ahlborg, Christian Jacobsson, Lotta Dellve

**Affiliations:** 1 School of Health Sciences, University of Borås, Borås, Sweden; 2 School of Technology and Health, KTH Royal Institute of Technology, Stockholm, Sweden; 3 Institute of Stress Medicine, Västra götalandsregionen, Gothenburg, Sweden; 4 Department of Public Health and Community Medicine, The Sahlgrenska Academy at the University of Gothenburg, Gothenburg, Sweden; 5 Department of Psychology, University of Gothenburg, Gothenburg, Sweden

**Keywords:** Leadership, Media, Stress, Healthcare management

## Abstract

**Background:**

Over the last decade healthcare management and managers have increasingly been in focus in public debate. The purpose of the present study was to gain a deeper understanding of how prolonged, unfavorable media focus can influence both the individual as a person and his or her managerial practice in the healthcare organization.

**Methods:**

In-depth interviews (n = 49) with 24 managers and their superiors, or subordinate human resources/information professionals, and partners were analyzed using a grounded theory approach.

**Results:**

The conceptual model explains how perceived *uncertainties related to the managerial role influence personification and its negative consequences*. The role ambiguities comprised challenges regarding the *separation of individual identity from the professional function*, the *interaction with intra-organizational support and political play*, and the *understanding and acceptance of roles in society.* A higher degree of uncertainty in role ambiguity increased both personification and the personal reaction to intense media pressure. Three types of reactions were related to the feeling of being infringed: *avoidance and narrow-mindedness*; *being hard on self*, *on subordinates*, *and/or family members*; and *resignation and dejection*. The results are discussed so as to elucidate the importance of support from others within the organization when under media scrutiny.

**Conclusions:**

The degree of personification seems to determine the personal consequences as well as the consequences for their managerial practice. Organizational support for managers appearing in the media would probably be beneficial for both the manager and the organization.

## Background

Over the last decade healthcare management and managers have increasingly been in focus in public debate. There seems, however, to be a lack of scientific evidence concerning the personal and organizational consequences for managers who are in the focus of negative media attention. Such “personification,” i.e., such increased focus, on the individual as a person rather than as a professional representative of the organization, is potentially harmful for both the manager and his or her organization. Using empirical data, this paper explores mechanisms of intense and unfavorable media attention and their consequences for managers in healthcare organizations.

### Increased focus on healthcare managers

Leadership in, and management of, public healthcare is a public concern and therefore an area where the media, the public, and politicians are expected to debate, investigate, and, where applicable, criticize managers’ decisions and strategies [1]. The complexity of leadership in the sector is increased by both mixed models of governance (political and managerial) and also the growing public expectation of transparent, open decision-making by public service management. New public management (NPM) has emphasized the personal responsibility of healthcare managers more strongly than previous management philosophies. The term “NPM” is an umbrella term for organizational reform methods that are strongly influenced by solutions derived from the private business sector based on trust in managers and markets, rather than in senior officials and the professions [2-4]. Forces that have driven these reforms include the need to balance the economy and the need for increased trust in public administration [5]. New public management strategies include decentralized responsibility, competition, marketization, and managerialism as guiding principles to improve economic control and efficiency of the public sector. In this transition work, the managers have the main responsibility and the outputs should be distinct, measurable, and transparent. In many countries, including Sweden, the demands for transparency in decision making and the economy are stronger in the public sector than in the private sector. The governance of healthcare service in Sweden has an increased dependency towards central authorities. Studies of governance have shown healthcare is far more centralized and formalized than other private and public organizations. However, the sector is also marked by “post-bureaucratic” forms of control that increased individualization [6]. Thus, healthcare services in Sweden have a high degree of centralized decision making and asking for directives, policies and rules, while the responsibility for the implementation of these are often highly decentralized to lower level managers [7]. Furthermore, Sweden’s laws prevent direct ministerial intervention on operational issues. This means the manager often faces the media on issues related to how political decisions have been operationalized into healthcare practice. Among Swedish citizens, the interest in healthcare and elderly care issues is increasing and is today among the highest ranking of public interest [8]. Although social media is becoming more important as a source of information, the primary source remains print and broadcast media. The media’s interest in the management of healthcare services has also increased. Today, not only political conflicts, but also mundane aspects of organizational life, have gained public attention through the media [9]. There are, however, no published studies investigating the prevalence of media attention with focus on managers of healthcare organizations. Preliminary results from our survey of all chief executives of municipal healthcare services, show that 50% had individual experience of being the spokesperson on occasions when there had been adverse media attention during the previous year [10].

In several cases, there has been a shift in media attention from structural aspects to aspects of personal agency in coverage of healthcare issues, which can put extraordinary pressure on the manager concerned. This can put extraordinary pressure on the manager being focused.

### Stress and pressures among healthcare managers

Healthcare organizations are expected to deliver high quality care, but managers in healthcare may have difficulties in forecasting fluctuations in demand and the key resources needed to achieve this aim [11]. In their work managing and developing healthcare, managers face a series of professional challenges as they experience high demands from higher managerial levels and from their own subordinates [12] as well as colleagues [13,14]. Their situation in this sense has been described as having to provide leadership during continuous change while trying to maintain trust and stability in their organization as well as sustain their own integrity [12]. Other elements of their situation that have been described are legitimacy-related pressures, loneliness, and ethical stress, as well as a lack of support from the organization [12,13].

The personal and organizational consequences of intense and personalized media attention need to be better understood. When the pressure gets too high, or ambitions are hindered, most individuals respond with some kind of stress reaction – of which there is a large variety. In their review of stress reactions, Schaufeli et al. [15] describe five different types of reactions. The first type constitutes affective reactions, such as anxiety, anger, apathy, and depressed mood. The second involves cognitive reactions, e.g., difficulties in decision making, or cognitive impairments. The third kind of reaction is physical, leading to, psychosomatic disorders or impairment of the immune system. The fourth type is behavioral, and includes hyperactivity and impulsivity. And lastly, the fifth kind of reaction is motivational, such as loss of enthusiasm, disillusionment, and demoralization. All of these, and probably especially the second and fifth type, may be detrimental to managerial work. Stress reactions also differ in their intensity and duration depending on the stressors involved, the coping skills of the individual, and the support (or lack of support) the individual gets. Stress can be easily overcome if it involves an occasional stressor. However, if there is prolonged exposure to a stressful stimulus, such as negative media focus, the individual’s resources for coping with, and adapting to, the situation may be insufficient, leading to an increased risk of developing chronic stress-related health problems [16].

Stress and pressures from intense media attention may have an impact on the managerial practice and the manager’s future handling of internal and external communication, as well as his or her future health and motivation to stay in his or her position. Recent research shows a high turnover rate among Swedish healthcare managers, with more than 40% quitting their job within 2 years [17].

To our knowledge, no previous empirical study has investigated mechanisms and consequences of intense negative media attention focused on individual healthcare managers and managerial practice. The aim of this study was to gain a deeper understanding of how prolonged, unfavorable media focus can influence the individual as a person and his or her managerial practice in the healthcare organization. The focus here is on the scrutiny which is brought to bear on managers as managers, rather than as individuals.

## Method

### Design

Our study design used grounded theory which is a qualitative approach and a systematic, explorative method. The aim of the grounded theory approach is to identify central processes and generate hypotheses and tentative conceptual models based on empirical data [18]. This approach was chosen because, to our knowledge, there is no research from a stress perspective focusing on the complex relationship between healthcare managers and intense critical media attention. We used qualitative interviews with managers and those around them (their own manager, a colleague, or their partner) to explore how negative media focus can affect the individual manager as a person and influence his or her managerial practice in a healthcare organization.

### Sample and data collection

The organizations were selected from different areas of Sweden to ensure that important similarities and differences were captured. We were careful to secure a study group which was balanced with regard to gender, geography, and the nature of their employing organizations (Table 1). Selection of managers being intensively and critically focused on in the media was primarily accomplished by contacting top managers of human resources departments (HR) in large healthcare organizations in different areas of Sweden. The inclusion criterion was that the managers had been the focus of prolonged critical media attention, including mention by name. The HR managers were informed about the study through a national network of HR managers, and were asked to provide examples of managers who had had a period of intense and critical media attention by reason of their professional work as managers within the last 3 years. Before passing on their names, the HR managers first asked the managers in this category if they were interested in participating in the study. All who were contacted agreed to participate. The managers were also asked for permission for their own superior, a colleague, and/or an important relative to be interviewed on the topic. This was in order to ensure a full description of managers’ reactions to the increased pressure. It also made it possible to describe reactions of which the manager being studied was unaware.

**Table 1:** Figures refer to number of interviewed persons (and extra interviews with focused persons in other roles)

**Case**	**Focused manager**	**Superior manager**	**HR or information specialist**	**Subordinate**	**Spouse**	**Total**
	**Male/female**					
Hospital healthcare	10/2	0 (2)	2	4 (3)	4	22 (5)
Municipal healthcare	7/5	1 (2)	2	2 (2)	1	18 (4)
*Total*	*24*	*1 (4)*	*4*	*6 (5)*	*5*	*40 (9)*

This study includes interviews with 24 managers who met the inclusion criteria (Table 1). All had top or middle management positions at a hospital organization (n = 12) or municipal healthcare organization (n = 12). The managers interviewed had all been the spokesperson to the media during either suspected mistreatment, or economic cut-backs with downsizing of clinics, or merging of hospitals or clinics, or privatization of certain healthcare services. Most of these managers were still working within the organization which had been the subject of the media attention under study, at the time of the first interview. Six of the managers under study left the organization for which they had been working within six months.

The study includes managers from 19 different healthcare organizations (10 hospitals and 9 municipal organizations). We deliberately sought to achieve a balanced sample of hospitals and municipal healthcare organizations of varying sizes. Although we did not decide in advance how many organizations to include in total or their exact distribution between different categories, our goal was to achieve conceptual saturation, with a wide range of scenarios included within the study, such that it could provide an adequate basis from which we could formulate a substantive empirically-grounded theory.

As well as the managers themselves, we interviewed a number of persons who had been involved as partners, subordinates, superior manager and colleagues. The managers themselves varied in the way in which they discussed their feelings and reactions, with some being more outspoken than others. Therefore the need for additional interviews varied. Furthermore, in some cases the managers themselves asked us to talk to someone else for additional information about the situation. Two spouses declined to participate in an interview.

Altogether, 40 individuals were interviewed. In nine cases, interviewees had double roles in the present study, both being the focus of a particular episode of media attention and being involved in an episode where another manager was the focus of such attention (see Table 1). Besides the 24 managers focused, five people were interviewed as superior managers, eleven as subordinate managers, four as HR or information professionals, and five as spouses or partners.

Qualitative open questions were used to encourage the interviewees to describe, in their own words, the process and strategies used to deal with the role of healthcare manager during episodes of close media attention. The interviews were conducted at a place where they felt secure and could talk freely. All interviews with the managers started with the same information about the study objective and the initial open question, “Have you experienced what it’s like to be the focus of media attention? If so, was it you, personally that came under scrutiny, and, in that case, how did you fell about that? were you personally focused and what was your experience of it?”

Different interview guides were then used for different categories, but all interviews covered the following themes:

• the episode during which the manager was the focus of media attention

• experience of supportive communication during the media focus

• reactions and actions of the management group

• their own reflections after the media focus ended

• family reactions and involvement

• scope for training to prepare managers for media attention.

The interviews lasted between 1 and 2 hours. Four managers were interviewed between two and four times within a 6-month period, in order to improve and deepen the descriptions. Interviews with subordinates and partners lasted about 1 hour. Prior to the interviews the participants were informed about confidentiality and their right to terminate their participation at any time. All participants gave informed consent in writing for their participation in the study, and the study was approved by the Ethics Committee of Gothenburg University.

### Analysis

The interviews were recorded and transcribed. The data were collected stepwise, simultaneously coded, and analyzed in line with the grounded theory approach [18]. The analysis in grounded theory studies comprises a rigorous and systematic process of coding and comparison of raw data, as well as the parallel use of theoretical memos and ideas [18]. The first step in coding aims to transform and conceptualize raw data into theoretical constructs. In other words, the researcher identifies and labels the pattern in raw data, repeatedly compares data and codes to identify differences and similarities, and sorts codes with the same content into categories. Each category is then further developed and related to its subcategories, dimensions, or properties. The last coding step aims to integrate and refine categories to form a dense and saturated theory. All steps include several discussions within the research group, with the purpose of challenging the interpretations and validating the preliminary categorization.

## Results

The results start with (a) a description of a typical scenario and continue with describing the (b) core-category and (c) categories and their sub-categories and dimensions.

### A typical scenario

A feature article. The article deals with the ongoing organizational change at a hospital focusing on the employees’ frustration, and possible risks related to patient safety and the working environment. One manager, named and pictured, is identified as responsible for the problem, in what is experienced as a rough, one-sided and simplified manner.

The manager experiences the situation as very pressing and unjust. A polarized situation develops among groups of healthcare professionals, media attention continues, and letters from the public on the matter begin to arrive. Members of the management team did not want to get involved. They hold their regular management team meetings, but neither the manager’s situation nor the media attention is on the agenda. In contacts with the media the manager has the feeling that he is already sentenced no matter what she says. This situation is maintained by articles and comments on social media about suspected irregularities and problems attributed to him as a person. He begins to avoid making important decisions, which negatively affects the ongoing development process at the hospital. Over time, the manager becomes more stressed and exhausted due to insomnia, lack of recovery and a lack of mutual trust in the organization. He turns to a few colleagues in whom he still has confidence and seeks support among his family and friends.

### Uncertainties related to the managerial role influence personification and its negative consequences

**Figure 1:**
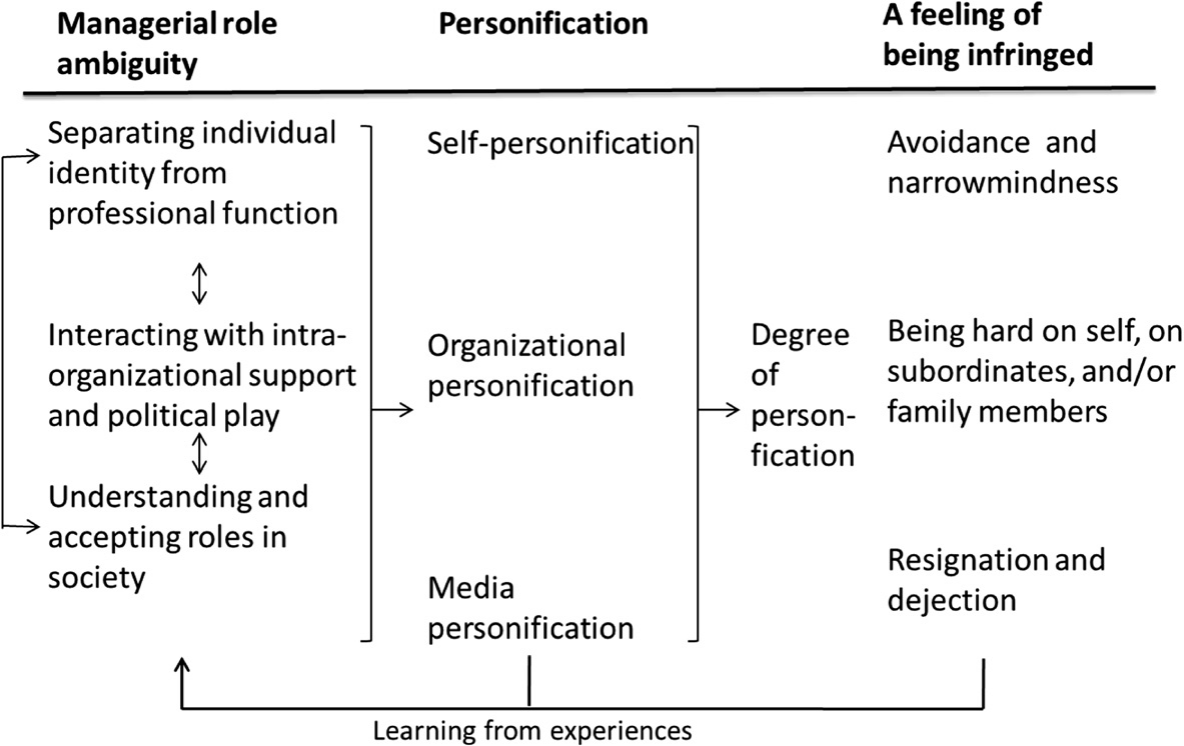
Uncertainties related to the managerial role influence personification and its negative consequences – a conceptual model of the dynamics leading to negative personal consequences from focusing on managers as persons while being subjected to media attention.

The conceptual model (Figure 1) explains how experienced *uncertainties related to the managerial role influence personification and its negative consequences* (core category). Personification is a construct describing how problems that arise within an organization are attributable to problems to do with the manager responsible and/or spokesperson. Role uncertainty was described as ambiguity related to conditions at three different levels: (1) *separating individual identity from professional function*, i.e., the extent to which the manager explains the situation in terms of personal deficiency; (2) *interaction with intra-organizational support and political play*, i.e., the trust, legitimacy, and support the manager experiences within the organization during episodes of media pressure; and (3) *the understanding and acceptance of roles in society*, i.e., realizing that a manager is a public person representing the organization in relation to society and the media*.*

The process of personification is a consequence of the ambiguities of managerial roles. In light of this, we identified three different levels of personification: (1) *self-personification*, i.e., the extent to which the manager explains the personification as something taking place within him or herself; (2) *organizational personification*, i.e., the degree to which the manager perceives people within the organization as blaming him or her and using him/her as a scapegoat; and (3) *media personification*, i.e., the extent to which the manager perceives that the media are focusing on him/her as a person rather than on the organizational problems that have triggered the media attention.

A high degree of role uncertainty increases both the degree of personification and the personally affronted reactions. Three types of reactions were identified: (1) *avoidance and narrow-mindedness*; (2) *being hard on one’s self, on subordinates, and/or family members*; and (3) *resignation and dejection*. These reactions influence the extent and nature of the organization’s ability to learn and change from the episode. The organization’s reaction to media focus is either functional and constructive or dysfunctional thus increasing negative personification.

### Managerial role ambiguity

Managers described uncertainties, such as managerial role ambiguity, which shaped their actions during media attention. These uncertainties may have been due to their perception of their own ability to handle their role during the time of media focus or to their understanding of strategies and actions needed. Further, they may be related to the kind of support they received. The central uncertainties were related to the following levels: the “person” described in the subcategory *separating individual identity from professional function*, the “organization” described in the subcategory *interacting with intra-organizational support and political play*, and “society” described in the subcategory *the understanding and acceptance of roles in society.* The level of uncertainty was also related to the degree of ambiguity at each level and their strengthening interactions, i.e., interactions between and within different levels. Certainties at one level may contribute to a more stable base despite uncertainty at another level, and vice versa. For instance, insecurity on the part of a manager could be reinforced by a lack of support from the management team.

#### Separating individual identity from professional function

This subcategory describes central uncertainties on the part of the managers themselves regarding their ability to separate their individual identity from their professional function that is, their ability to view the criticism as directed towards their professional role and not at themselves as individuals. During the interviews, all managers exhibited awareness at an intellectual level of the importance of separating person from function.

Manager: *Managers that can find themselves in delicate situations need to be confident individuals that are always capable of separating their functional role from who they are as a person…it’s a survival strategy.*

Manager: *… when they question my actions as a person, it’s an attack on the whole system, not me.*

Manager*: I need to stand for my actions in my capacity as a private individual… It’s a strange situation - especially when you’re on TV and your child wonders why you’re on TV… You are not in your professional roll then…that’s me the person.*

However, the managers also described reactions of increased heart rate, panic attacks, irritability, and insomnia, which may be explained by severe difficulties or inability to do this consistently in practice. Further, it may be more challenging for managers who strongly identify with their managerial objectives to accomplish this separation. All participating managers identified to varying degrees with the objective of improving the healthcare organization. They described this in terms of an action oriented ambition to implement organizational changes in order to improve efficiency and quality of care. The managers described this goal as a precondition for accepting their position.

#### *Interacting with intra-organizational support and political play*

This subcategory describes intra-organizational dynamics and support systems of importance for the managers’ experience of legitimacy within the organization in times of media pressure. The managers described their legitimacy, given to them by powerful intra-organizational groups, especially representatives of stronger professional groups, e.g., physicians, as important in giving them certainty in their managerial role when under scrutiny from the media Also, for those managers working closer to the political system the trust experienced interactions with politicians was important in generating confidence when under media pressure. The managers related how uncertainty about the organization’s history with regard to media relations and its current willingness to handle media focus influenced the basis for their managerial role. They described how, when expectations of support from the organization were not met, they felt insecure and uncertain about the kind of support they could expect in the future. This influenced their reaction to media pressure. For example, they became more narrow-minded and avoidant.

Challenges presented by negative media attention could be seen as an issue for the entire organization or as an issue for the manager. The degree to which a given organization was prepared and ready to deal with such internal and external challenges was often described as limited. In these cases, the managers were not aware of any organizational plans for how to handle media focus. The organization’s policies for, and practices with regard to, managing this type of situation were insufficient in the sense that they did not provide guidance as to which strategy might be the best for both the organization and the exposed manager. There seemed to be a tendency, often based on tradition, to continue to let the individual manager answer the questions posed by the media. This strategy seemed to strengthen the individualization of the problem by the organization, and successively led to more focus on the person rather than on the organization, with the manager as its representative.

Manager: *If the function is extremely well defined, then it is also much easier to relate to this as my function, not my person when I’m portrayed…*

Strategies for supportive actions by top management, the management group concerned, and specialist functions within the organization, e.g., information/communication professionals, seemed to be limited and in some cases non-existent:

Manager: *There was a smear campaign against me fairly early in the hospital.*

Senior manager: *… I think it's a combination of a number of different variables, it involves both her way of addressing these issues and her determination. She has not always been great at communicating what she has done to others, there may have been […] a lack of understanding of certain things in the organization, and it might also be said that she is unaccustomed to working directly on behalf of the politicians. Even though I am in some sense an intermediary, a link, it is still the case that a manager at this level pretty much works directly with politicians, and if you are not quite used to doing that, things don’t always turn out for the best.*

#### *The understanding and acceptance of roles in society*

Managers’ understanding and acceptance of their role as managers in public healthcare, in a societal perspective, affected their confidence in their managerial role. Having an awareness and acceptance of their role in society and also of the role and power of the media gave a firmer managerial base and, consequently, a greater ease of contact with the media. Managers who described themselves as more confident in this respect could give matter of fact answers more easily, which seemed to invite less personification and negative reactions from the media. A lack of knowledge about their role in a societal perspective, on the other hand, was associated with lower confidence in contact with the media and defensive managerial approaches, such as withholding information, being unavailable, or defending themselves rather than addressing the issues which concerned the organization.

Manager*: We have spent a lot of time and resources getting our people properly trained as communication experts. This has allowed us to be better at using our web page to deliver news – when we post an exciting story the print and broadcast media tend to run with it rather than arriving at the story in a roundabout way – the first scoop will contain our wording of the story. We have invested a lot on properly trained communication experts… we will be able to better provide information and to post news [… ] When we post exciting news, the media takes it, instead of it possibly becoming a detour, it will be our words written in the first scoop…*

Manager: *"You don’t sell a lot of papers on catchy articles –you need a bit of misery as well! …and we need to work harder to make sure that there isn’t such an awful lot of misery …"*

Some managers also expressed an awareness and acceptance of the media interest due to the type of healthcare they were responsible for. For example, being a manager of emergency care was described as attracting more media attention compared to less exposed units of a hospital. Managers of emergency care units, said that they had to accept being the object of media focus more often, because the general public can more easily relate to the importance of a well-working emergency healthcare with its more obvious bearing on life or death decisions and situations.

### Personification

The extent of the personification and the amount learnt from being focused on by the media were both influenced by the managers’ degree of confidence about the managerial role during periods of strong pressure. The combination of individual, organizational, and media personification further affected the severity of managers’ feeling of being personally affronted and the reactions related to this. Where personification was used by the media only, the damage was not as extensive as when he manager him or herself and/or important parties in the organization also contributed to the personification process. The personification processes could start within one subcategory, and then affect the other subcategories in any direction, to either strengthen or weaken the focus on the manager as a person.

#### *Self-personification*

The managers’ own ability to separate their personal self from their professional role seemed to determine the degree to which they identified with what was said about them in the media. Where a manager took the media focus personally, even if the text was directed towards organizational features, this seemed to enhance his or her negative reactions:

Manager: *That’s the role you have, that’s the role that is exposed.*

Partner: *… when something would be presented, or brought forward … he was very exposed. He had the ability not to take it personally … he could see, intellectually that it was about his roll … that it wasn’t him as a person that was being attacked, but his role.*

#### Organizational personification

Personification in situations of media focus on an individual manager can start from within the organization. In such cases, the personification can be the result of internal forces to change positions of power among professional groups and/or persons within the organization. It can also be the result of efforts to protect the organization’s reputation in society. In order to maintain that the manager in question as an individual is to blame if the media and/or the person themselves have already personified the problem.

Manager: *There was not that common humanity … there was more interest in protecting the hospital’s reputation and they really didn’t care a damn about me.*

Colleague: *When the person is exposed to pressure, the organization withdraws from the individual.*

Partner: *I felt that he was pushed in front of his boss. … I don’t know if it is unique to the hospital to find scapegoats all the time, but I experienced enough to see that my husband became the scapegoat for too much … as a person.*

Senior manager: *I thought I might go to a meeting to calm things down and lend support, I would have gladly done this but I came to the conclusion that I should not do it because I was afraid the focus would be moved to me. This is a tough business at times, if you want to be a manager, you have to do it yourself. It’s the manager’s lot in some in a sense of being alone.*

#### *Media personification*

The interviews and documentation from this study show that the managers experienced that media tended to focus on the individual manager, rather than on the organization. The media seemed to be more likely to negatively personify the managers in cases where they expressed ambiguity. Also, allowing comments from the readers (via the social media) that personified the manager, in connection with web-based articles, further increased personification of the manager.

Manager: *They don’t love what I do, so they probably don’t love me either.*

An example of unfavorable personification by the media was a situation, described by one of the interviewees, when television cameras recorded a manager scratching her head before answering the reporter’s questions. Afterwards, in the report, this scene was replayed several times over as a way to suggest that this person had difficulties delivering a straight answer. For several weeks, television showed this same scene, of the manager scratching her head, when reporting on the case. Other examples were newspapers publishing the names and photos of the managers, presenting them as “organizational problems.”

### A feeling of being infringed

The sense of personal affront that the managers experienced as a result of personification arose from the managers themselves, from the organization, and/or from the media. The resulting personal reactions were related to the degree that they perceived themselves the target of unjust media focus. Personification by more than one of these three sources seemed to increase the perceived sense of affront in an additive way.

The managers described their personal reactions in relation to their personal decision to take the role of manager and the responsibility that goes with it. The managers had entered the managerial position in the healthcare organization because they were driven to succeed. They had an idea of what they wanted and how they could influence change within the organization. In the interviews, they said that much of their initial energy had been lost. One reason for this was the media pressure. They said they felt that their energy loss was related to the degree of personification, and the lack of support that they had received when under media focus. Internal support in the form of discussions with top management, communicative support from an information service, or support from their own managerial team served to reduce the pressure and, consequently, the loss of energy. Having the ability to separate personal identity from professional function seemed to have similar mitigating effects. The perceived degree of feeling assaulted to a feeling of being infringed influenced the long-term consequences in terms of personal learning from the experience, and, further, the perception of uncertainties related to the managerial base. Three types of reactions were identified and are described below.

#### *Avoidance and narrow-mindedness*

One of the reactions that the managers described was the tendency to withdraw and communicate with only a few people or just keep things to themselves. Managers who were avoidant and limited their contacts perceived a limited general trust in people in the organization, which appeared to be related to the organization’s communication and support systems. When the media focus was started by internal actions, when people within the organization used the media as a way to achieve their own goals, distrust was increased. Those managers who reacted in this manner narrowed down the group of people they trusted to a handful during the period of media focus. Thus, in these cases the management teams were in practice limited to a few selected individuals.

Some interviewees related how the experience of feeling under attack and personally affronted, as the following quotes show:

Manager: *I have to constantly make sure I have people around me whom I know.*

Manager: *… you become more concerned about integrity.*

Colleague: *… The manager chose to create a smaller management group…*

Manager: *You learn to distinguish between friends and enemies … who you can trust and who you cannot trust.*

A typical example of being avoidant was a manager not making decisions or procrastinating as a consequence of being afraid of more resistance and publicity. The reason for avoiding making decisions that might be controversial, and, as a result, evoking the media’s interest, was a temporary “media fatigue” which made the manager anxious to maintain the energy he or she had left. The more controversial managerial decisions were postponed till later, when the media focus would have decreased.

#### *Being hard on one’s self, on subordinates, and/or family members*

The study includes examples of how managers claimed to be able to handle the pressure and the psychological impact of the media focus on their own. These managers viewed media attention as something that comes with the job and believed that they were expected to cope with it on their own. They thought that to that they felt affronted and ask for help was to show vulnerability and weakness. Similarly, they seemed to transfer these expectations of “being tough in relation to media pressure to their subordinates, colleagues, and family.

Manager: *… I had panic disorder, for example, but I learned how to handle that myself …*

Partner: *He became angry for the slightest little thing … He was angry with the kids … I didn’t recognize his behavior”*

An example of toughness towards subordinates was not offering them support when they talked about having problems during times of intense media focus involving themselves. These subordinates registered a change in their manager’s qualities and character, from being supportive to being unsupportive and expecting everyone to mind their own business.

#### *Resignation and dejection*

Resignation and dejection were described in cases of managerial exposure to more extensive and repeated negative media focus and when the managers’ general faith in their ability to cope with the situation and the expected support had undergone a more profound change. Furthermore, the managers’ general confidence in the organization had also declined. The managers concerned described not being sure about what to expect. They said that the number of people they trusted had decreased. This, in turn, was related to apathy and lethargy, which sometimes led to avoidance of difficult decisions.

Coworker: *She was not as energetic as usual.*

Partner: *He was hurt … he seemed depressed…quieter …*

He was heavily influenced by it. … I could almost feel that he was depressed … maybe too strong an expression, but he was depressed during that period … he was not feeling well … he was not.

Manager: *I have become more cautious now … I’ve learned something … think first and then act …*

Manager: *It became everything…*

Partner: *Someone important was hurt… everyone in the family has been affected..*

Partner: *She has become more and more depressed and withdrawn…with a rising disinclination to go to work..*

Spouses described how their partners withdrew and seemed resigned during the period of negative media exposure, leading a less active social life, being alone more often, and being less talkative. There were also cases where managers admitted to increasing their alcohol consumption, as a way to handle their anxiety and sense of personal affront.

## Discussion

The results describe a conceptual model of how individuals as well their organizations in interaction with the media contribute to role uncertainness. The managers’ experience of being fringed as a result of personification was found to arise from the managers themselves, the organization, or the media. The degree of personification seemed to determine the personal consequences as well as the consequences for their managerial practice. Most managers showed that they knew about the importance of separating function from person in their job, but the results from the analysis of the in-depth interviews highlight the difficulty doing just that when under intense media hit. One explanation for this may be the strong driving force to improve the organization they were in charge of. All those interviewed said that they already had such feelings when they started to work in their present position as manager. The personal indignity of being made the victim of a negative personification can therefore be seen in relation to all the efforts they had made to fulfill their inner mission of making meaningful improvements in the healthcare organization they were responsible for.

The results can be related to theories of work identity. Managers’ work identity is influenced by combined organizational and managerial processes [19]. Managers’ work identity processes have been described as a struggle and as having many conflicting expectations [20]. A manifestation of this interest in managerial identity is provision of management training programs supporting personal growth, personal development, and self-knowledge. This focus on personal development disregards the importance of consider intra-organizational interactions and its influences on the manager. Managers’ work identity development is dependent on their interaction with the organization [21]. Andersson (2005) describes the struggle between “being you” and being a “representative role model” as a manager within the organization. He states that the manager’s work identity cannot be seen without this ongoing process. It is a part of the whole [22].

The ideas of NPM may have the effect of creating and strengthening driving forces of individualization and personification of organizational performance. There may be individuals with an inner goal who are being recruited for their individual strength and sense of mission. This may contribute to passivity in other actors, who also have responsibility for decisions and performance in the organization. This situation may carry an increased risk of the organization becoming dysfunctional if the manager in question has a personal breakdown due to overwhelming media pressure. Some of the interviewees described how a situation much like this had developed during the process surrounding aversive media coverage. The media-related pressure may be one explanation for some managers’ perceived indignity and loss of energy, as well as for the perceived general uncertainty of their managerial base; in other words, in their case, the media focus triggers of general experience of role ambiguity.

The reactions to increased pressure in the presented model are to some extent connected to how the burnout phenomenon is described in research. According to Maslach et al. (2001), burnout is a prolonged response to chronic emotional and interpersonal stressors at work. It is defined as a three-dimensional concept consisting of exhaustion, cynicism, and feelings of inefficacy [23]. The three reactions to pressure in the present model could be interpreted as connected to the burnout process. Being avoidant and narrow-minded could be seen as attempts to take control and thus uphold personal efficacy despite the pressure. Being hard on one’s self, on subordinates, and on the family might be interpreted as being related to cynicism. Trying to establish emotional and cognitive distance between one’s self and the pressuring situation is an attempt to make the demands more manageable. Lastly, resignation and dejection could be seen as being connected to exhaustion, the basic indicator of burnout [23]. Being less energetic is a similar response to stress exposure as being exhausted. Therefore, being exposed to negative media focus with elements of personification can increase the risk of burnout in some managers.

How can we understand the wider effect of media focus on the individual managers and the organizations? To our knowledge, no previous study has been performed on the effects and consequences for the individual managers subjected to negative media coverage. However, there are a few studies investigating the effects on organizations see, e.g. [24] and a large amount of literature focuses the effects of the media and external communication in general. It is important to acknowledge the dynamics involved in the processes to better prepare and support managers and their organizations. Kjaer & Slatta (2007) point to the importance of deconstructing “the black box” of the media to get a more nuanced appreciation of media work in order to avoid gross oversimplifications (in relation to both the media, and managers and organizations). Using different theoretical perspectives Kjaer (2009) has, summarized dynamics that may create an organizing role for media focus. According to his review, media focus may organize the dynamics of reputations, identities, and authority as well as ideas and practices within the focused organization [25]. On the other hand, organizations’ interest in high media visibility has increased [26]. Chen & Meindl (1991) argued that the personifications of managers are enhanced by formats and routines of media production and practical constraints faced by journalists [27]. Grafström et al. (2013) claim that the interaction between an increased role for media coverage and media’s role as a “moral court” support increasing levels of personification. Furthermore, according to media logic, people are more interesting to the public than are organizations. This can lead to managers becoming heroes or being vilified in this dynamic between organizations’ possible gains or losses related to media exposure. However, the role of the media depends on the media’s impact on the public and the type of content presented. Today healthcare issues are among the highest ranking areas of public interest in the national media [8]. In local media, the impact on the readers is higher if the information is locally relevant, such as healthcare and care for the elderly [28]. The role of media as a normative “moral court” increases when the topics covered are complex value-laden issues, such as healthcare service [26]. The results from the present study contribute to this area of research by suggesting a conceptual model of how the dynamics of ambiguity in organizational and personal bases interact with personification processes and the resulting consequences for the individual manager. The individual consequences also have a fairly large impact on managerial and organizational media contacts.

The intra-organizational demands on managers may be understood as being due to (1) the ideas of NPM for replacing the old philosophy of professional control; (2) a gap between leadership and operational levels due to NPM control systems being described in general (rather than professional) terms; and (3) interorganizational conflicts of interest. Managers in the present study described how media focus on the managers and the negative personification processes started from an intra-organizational quagmire of conflict. Results from a recent study [25] also highlight the fact that the media has become an important instrument for employees of healthcare organizations to increase their influence on decisions [29]. In several cases in the present study, the employees of healthcare services had provided information to the media. Employees are aware of the power they potentially wield if they use the requirement for transparency that should characterize public organizations in Sweden [30] Several previous studies describe the impact of bottom-up processes in an organization, where professional groups (mainly doctors) used both formal and informal communication channels [14,29]. By interacting within the professional groups, the media, and political opposition, they obtained wide informal power [31]. Conclusions from these studies were that healthcare leadership should include a deeper awareness of these multiple interests and prioritize time for internal communication processes. In a similar way the present study describes how the personification of problems increased through organizational political games, with strong professional groups and/or political opposition influencing the managers’ confidence and their perceived legitimacy and legitimacy in their role during media pressure.

Thus, leadership ideals are related to administrative skills with a focus on the economy as well as external legitimacy, in terms of familiarity with the operative work and sustaining good external relationships through communication [32].

## Conclusions

In conclusion, the degree of personification seems to determine the personal consequences as well as the consequences for their managerial practice. Organizational support to managers during media communication would probably be beneficial for the manager and the organization.

### Methodological limitations

In the present study, we aimed to develop a model of healthcare managers’ experiences and reactions during extensive negative media focus. The model is empirically based on 24 cases, drawn from a total of 49 interviews. This could be considered a large qualitative or a small sample; however, the interview data were replicated several times, resulting in data saturation. Since the managers themselves were asked if the media focus was unfavorable the inclusion criteria were based on their subjective choice. This is a limitation because other managers, not included here, could have been the focus of even greater negative media coverage, according to more objective criteria.

#### Suggestions for future research

One of the inclusion criteria for the present study was that the managers should have been negatively exposed >by name in the media. This may be seen as too restrictive since being the focus of negative media coverage may not always be needed to evoke similar experiences and reactions to those presented in the model. Individuals who work in the organizations in focus may react in similar ways even if they are not the direct subject of media attention. Such cases should be explored in future studies.

### Practical implications

This study, though based on a small sample may contribute to normalizing the reactions described by the interviewees. Recognizing that these kinds of reactions are normal in this particular situation will hopefully contribute to proactive development of better strategies at the individual and organizational level to cope with such pressure. Such improvements would probably decrease the risk of personification in the media and its negative consequences.

## Competing interest

There are no competing interests to declare, either financial or non-financial.

## Authors’ contributions

MWW participated in the design of the study, conducted the majority of the interviews, analyzed and interpreted data and drafted the manuscript. CJ conducted interviews, participated in the interpretation of data and were involved in drafting the manuscript. GA participated in the design of the study, the interpretation of data and critically revised the manuscript. LD participated in the design of the study, conducted interviews, analyzed and interpreted data and drafted the manuscript. All authors read and approved the final version.

## Authors’ information

MWW (PhD-student) and CJ (assoc professor) are psychologists and has many years of experience of management consultancy. GA is a MD, an associate professor in occupational medicine and are director of a clinical and research institute for stress medicine. LD is a full-time professor in health science with a focus on leadership and organizing of healthcare service.

## Pre-publication history

The pre-publication history for this paper can be accessed here:

http://www.biomedcentral.com/1472-6963/14/8/prepub
